# Combined High-Pressure and Multiquantum NMR and Molecular Simulation Propose a Role for N-Terminal Salt Bridges in Amyloid-Beta

**DOI:** 10.1021/acs.jpclett.1c02595

**Published:** 2021-10-07

**Authors:** Sahithya Phani Babu Vemulapalli, Stefan Becker, Christian Griesinger, Nasrollah Rezaei-Ghaleh

**Affiliations:** † Department of NMR-based Structural Biology, Max Planck Institute for Biophysical Chemistry, Göttingen 37077, Germany; ‡ Institute for Chemistry and Biology of the Marine Environment, University of Oldenburg, Oldenburg 26129, Germany; § Department of Neurology, University Medical Center Göttingen, Göttingen 37075, Germany; ∥ Institute for Physical Biology, Heinrich Heine University, Düsseldorf 40225, Germany

## Abstract

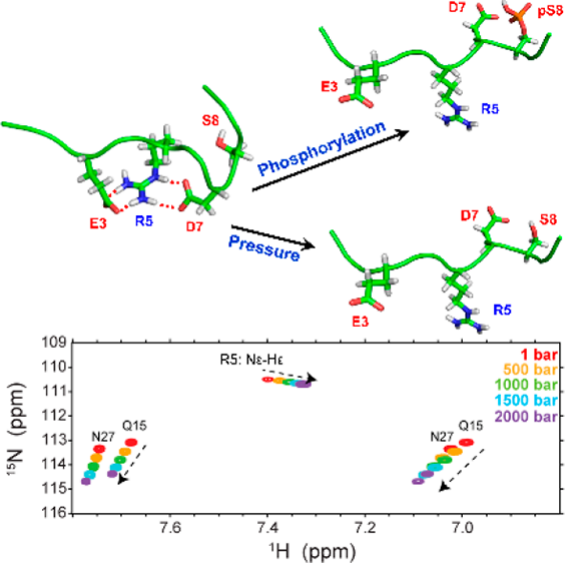

Several lines of evidence point to the important role of the N-terminal region of amyloid-beta (Aβ) peptide in its toxic aggregation in Alzheimer’s disease (AD). It is known that charge-altering modifications such as Ser8 phosphorylation promote Aβ fibrillar aggregation. In this Letter, we combine high-pressure NMR, multiquantum chemical exchange saturation transfer (MQ-CEST) NMR, and microseconds-long molecular dynamics simulation and provide evidence of the presence of several salt bridges between Arg5 and its nearby negatively charged residues, in particular, Asp7 and Glu3. The presence of these salt bridges is correlated with less extended structures in the N-terminal region of Aβ. Through density functional theory calculations, we demonstrate how the introduction of negatively charged phosphoserine 8 influences the network of adjacent salt bridges in Aβ and favors more extended N-terminal structures. Our data propose a structural mechanism for the Ser8-phosphorylation-promoted Aβ aggregation and define the N-terminal salt bridges as potential targets for anti-AD drug design.

Amyloid-beta (Aβ) aggregation into senile plaques is a neuropathological hallmark of Alzheimer’s disease (AD).^1^ Aβ aggregation is widely regarded as the initial event in AD pathogenesis and is therefore a suitable target for anti-AD drug development.^2^ The rational design of aggregation inhibitors demands a detailed understanding of the mechanism of Aβ aggregation, especially in the early steps when the more toxic nonfibrillar aggregates of Aβ are formed.^3,4^

Several atomic-resolution structures of Aβ fibrils have been published in recent years, providing a wealth of information about the end state of their aggregation pathway.^5−7^ In addition, the intrinsically disordered monomeric state of Aβ has been characterized in detail.^8−11^ Despite extensive effort, however, the structural knowledge about the intermediate states of Aβ along the aggregation pathway is relatively limited.

The N-terminal region of Aβ, that is, residues 1–10, is the host for several AD-related mutations, such as A2V, H6R, and D7N mutations,^12−14^ and modifications, such as N-terminal truncation, S8 phosphorylation, and Y10 nitration,^15−17^ which alter its aggregation propensity. Because this region appears to be relatively unstructured in Aβ fibrils,^18^ the altered aggregation behavior of Aβ upon its N-terminal modifications suggests that the N-terminal region of Aβ may play an important role in the intermediate stages of Aβ aggregation. Previous studies point to the role of the N-terminal region of Aβ in an oligomer-stabilizing network of interactions.^19^ In line with this hypothesis, proline mutagenesis studies suggest that an N-terminal β-strand of Aβ controls the partitioning between oligomer and protofibril aggregation.^20^

Some recent structural models of Aβ fibrils demonstrate the involvement of N-terminal charged residues in local or long-range and intra- or intermolecular salt bridges;^5,21,22^ however, there is little, if any, experimental support for the presence of N-terminal salt bridges in Aβ monomers. The N-terminal region of Aβ is relatively rich in both positively and negatively charged residues. The potential network of electrostatic interactions makes this region amenable for high-pressure NMR studies because of the considerable volume decrease associated with charge separation.^23^ High-pressure NMR studies of several amyloidogenic proteins have proven useful in detecting and structurally characterizing their conformational substates and correlating them with their distinct aggregation propensities.^24−28^ Here we study the structural dynamics of Aβ dependent on pressure with a focus on its N-terminal region. Combining NMR with the analysis of a previously published MD trajectory,^29^ the presence of N-terminal salt bridges is demonstrated. Using density functional theory (DFT) calculations, we propose a mechanism for the S8-phosphorylation-induced enhancement of Aβ aggregation.

First, NMR spectra of Aβ peptides were measured at different pressure levels ranging from ambient pressure to ∼2000 bar. The ^15^N,^1^H correlation peaks were generally shifted by pressure change, especially in the HN chemical shift dimension, which is particularly sensitive to the hydrogen-bond interaction with the network of surrounding water molecules (Figure 1a). The direction of peak displacement indicates further deshielding at higher pressures and suggests a relative enhancement of the peptide–water hydrogen bond interactions. In ^13^C,^1^H correlation spectra, the pressure-induced peak shifts were less marked, but several peaks, in particular, N- and C-terminal residues, Asp1 and Val40, and charged residues, Arg5, His6, His14, and Lys16, exhibited a prominent displacement in both the Hα and Cα chemical shift dimensions (Figure 1b). In addition, the HCO planes of the HNCO spectra revealed the pressure sensitivity of CO chemical shifts in many peaks (Figure S1). Similar spectral changes upon pressure rise were also observed in Aβ42, an Aβ variant with an identical amino acid sequence except for two additional residues in the C-terminus (data not shown).

**Figure 1:**
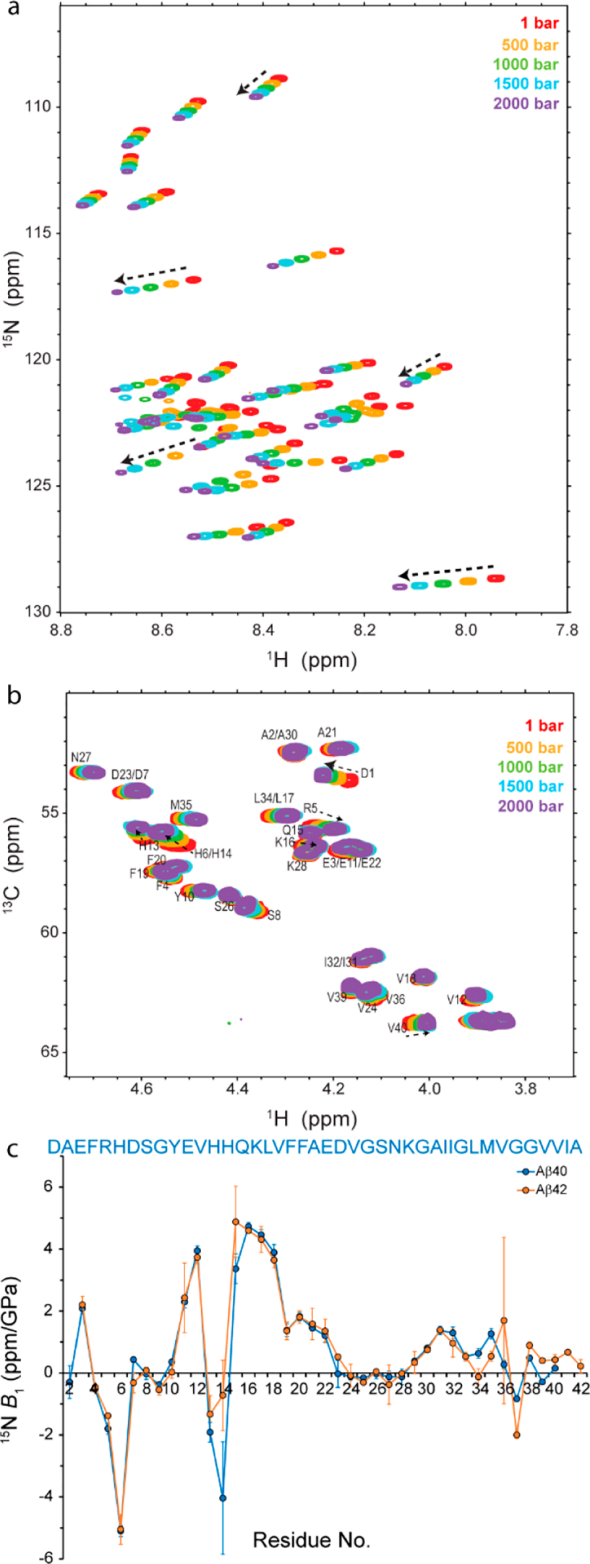
Pressure dependence of NMR spectra of Aβ40. (a) ^15^N,^1^H and (b) ^13^C,^1^H HSQC spectra obtained at 1, 500, 1000, 1500, and 2000 bar. Note the direction of the peak displacement, which is highlighted by dashed arrows, mainly for N-terminal residues. In panel c, the first-order (*B*_1_) pressure coefficients of the backbone amide nitrogen over the sequence of Aβ40 (blue) and Aβ42 (orange) peptides are shown.

The effect of pressure on the chemical shifts of protein amides can be analyzed in terms of first- and second-order coefficients, *B*_1_ and *B*_2_, obtained through fitting to a second-order Taylor expansion of chemical shift perturbations with respect to pressure changes. (See the Supplementary Methods.) After correction for the pressure effects observed in random-coil model peptides, the linear or first-order *B*_1_ pressure coefficients captured most of the pressure effects. As shown in Figure 1c (and Figure S2), the overall profiles of amide nitrogen (and proton) *B*_1_ coefficients were highly similar over Aβ40 and Aβ42 sequences. Notably, residues Glu3, Glu11-Val12, and Gln15-Glu22 showed relatively large positive nitrogen *B*_1_ values, whereas residues Arg5-His6 and His13-His14 had relatively large negative nitrogen *B*_1_ values. The intervening regions Asp7-Tyr10 and Asp23-Gly29 showed near-zero nitrogen *B*_1_ values, indicating that the pressure dependence of their amide nitrogen chemical shifts was indistinguishable from that of random-coil peptides. Similarly, the amide proton *B*_1_ profiles were characterized by marked elevated *B*_1_ regions at residues Arg5-Ser8 and His13-Phe19 (Figure S2). In both amide nitrogen and proton *B*_1_ profiles, the *B*_1_ values in the C-terminal region were generally small. Taken together, the pressure coefficients of amide chemical shifts point to the presence of pressure-sensitive structural elements in the N-terminal region of Aβ.

NMR chemical shifts are sensitive probes of conformational dynamics in proteins.^30^ Using the set of backbone chemical shifts CO, Cα, N, HN, and Hα (plus Cβ), we predicted distinct structural motifs in Aβ dependent on pressure.^31^ As shown in Figure 2, the calculated probability for the extended strand formation was increased by pressure rise, mainly at the expense of the probability for the compact loop structure, which was reduced. Then, the pressure dependence of the Aβ backbone flexibility was monitored through the random-coil index (RCI)-based squared order parameter (*S*^2^), a parameter qualitatively representing the heterogeneity of the conformational ensemble of Aβ. Except for residues Ser8-Glu11 and Asp23-Gly25, which showed less mobility at higher pressure levels, the pressure rise led to a general enhancement in the backbone mobility of Aβ40, in particular, in residues Arg5-His6 and His13-His14-Gln15-Lys16 and, to lower extent, in the C-terminal region between Asn27 and Met35 (Figure S3). Similar changes were observed for Aβ42 (Figure S4). Together, the chemical shift data suggest that the increased pressure disrupted local interactions in Aβ and consequently led to more extended conformations and a larger backbone mobility. Because the Aβ40 concentration of ∼70 μM used in these experiments was well below the threshold concentration for Aβ40 oligomerization (∼120 μM at ambient pressure),^32^ the disrupted interactions are expected to be largely intramolecular, although a small contribution of intermolecular interactions cannot be strictly excluded. It is also noteworthy that the NMR chemical shifts of charge-bearing residues, in particular, histidines, and their adjacent residues are particularly sensitive to their charge state; therefore, the NMR chemical shift data alone are not sufficient to support the previously mentioned hypothesis.

**Figure 2:**
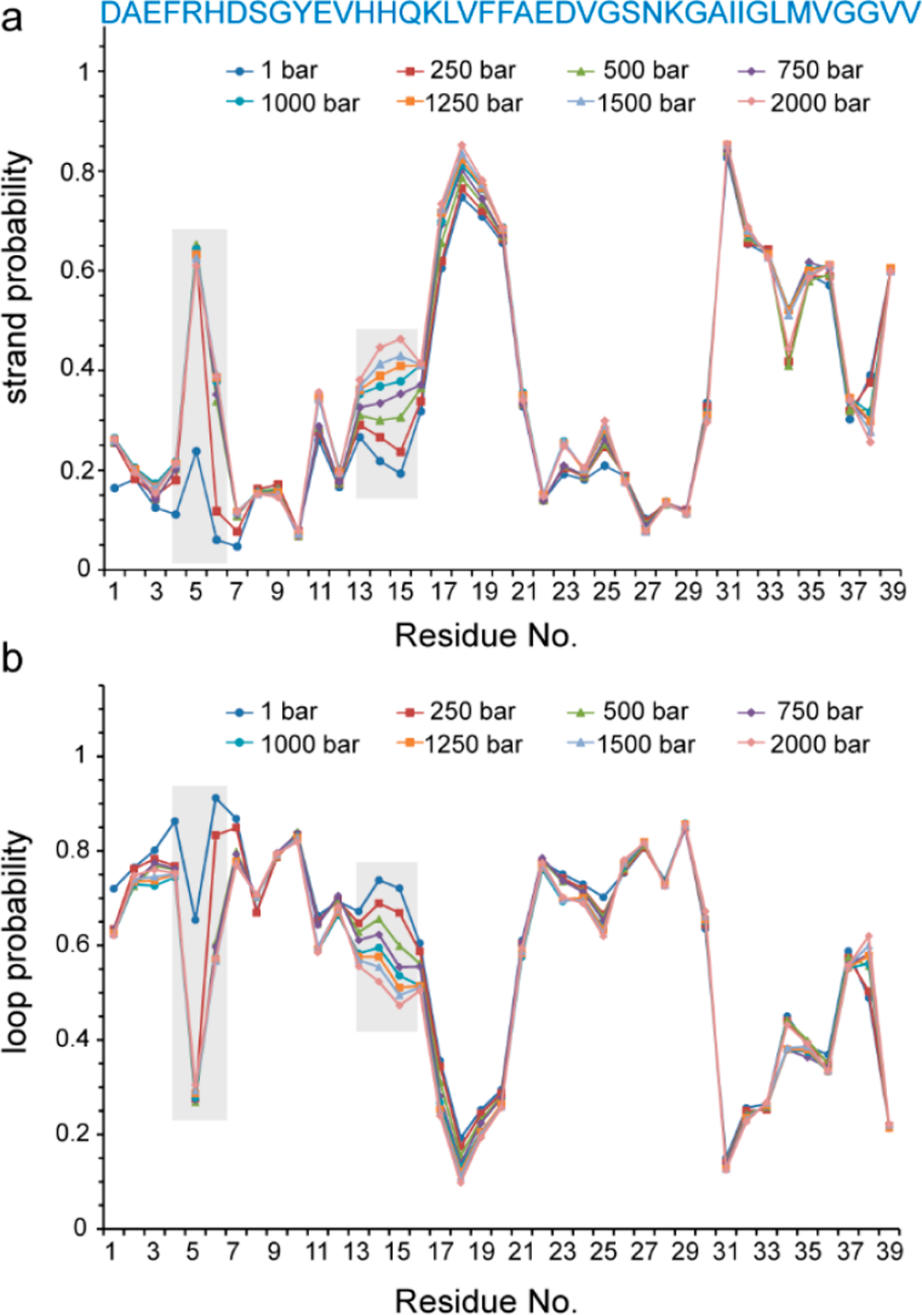
Pressure dependence of the structural ensemble of Aβ40. Residue-specific probability for the formation of (a) strand and (b) loop structures, calculated from backbone (CO, Cα, Cβ, N, HN, Hα) chemical shifts. Residues R5-H6 and H13-K16 exhibit a bigger probability of strand formation at higher pressures, largely at the expense of the probability of loop formation (shaded boxes).

A potential source of pressure sensitivity in the structure of proteins is salt bridges, where the volume decrease upon electrostriction of solvent molecules by separated charges favors salt bridge disruption at high pressures.^23^ The possibility of pressure-induced salt bridge disruption in Aβ is further highlighted by the observation that the structural propensities and backbone mobility are most prominently affected in the highly charged regions, Arg5-His6 and His13-Lys16 (see above). Interestingly, in the case of Arg5, the high-pressure NMR data provided an experimental support for the presence of a salt bridge and its sensitivity to high pressure. As shown in Figure 3a, upon increasing pressure, the Arg5 side-chain (Hε-Nε) peak in the ^15^N,^1^H HSQC spectra was displaced. Unlike the backbone (and Gln15 and Asn27 side chain) ^15^N,^1^H correlation peaks, the pressure coefficient of the Hε chemical shift was negative; that is, it decreased by pressure. This indicates less deshielding at higher pressure levels and is consistent with the pressure-induced disruption of an Arg5-based salt bridge. Furthermore, the intensity of the Hε-Nε peak was increased by pressure (Figure 3a,c). A similar proton chemical shift and intensity changes were observed in the Arg5 side-chain (Hε-Cζ) peak in the HCO plane of the HNCO spectra (Figure 3b). The direction of changes in Hε, Cζ, and Nε chemical shifts (decrease in Hε and Cζ and increase in Nε chemical shifts) is consistent with the expected pressure-induced disruption of the potential salt bridges involving the Arg5 side chain, as demonstrated by the DFT-based prediction of chemical shifts in a model pentapeptide (Glu-Gly-Arg-Gly-Asp) mimicking the salt bridge between Arg5 and Glu3/Asp7 (Figure S8, Table S1). We propose that the intensity gain of the Arg5 side chain is caused by a pressure-induced drop in the population of the Arg5-based salt bridge and its consequent reduction in the exchange-mediated relaxation rates. Interestingly, the pressure-induced increase in the Arg5 Hε-Cζ peak intensity was accompanied by a considerable enhancement of its satellite peak (Figure 3b), based on which we speculate that the pressure-induced disruption of the Arg5-salt bridge allows populating an otherwise minor N-terminal conformation in Aβ40. Similar changes in the Arg5 side-chain peak intensities were observed for Aβ42 (Figure S5).

**Figure 3:**
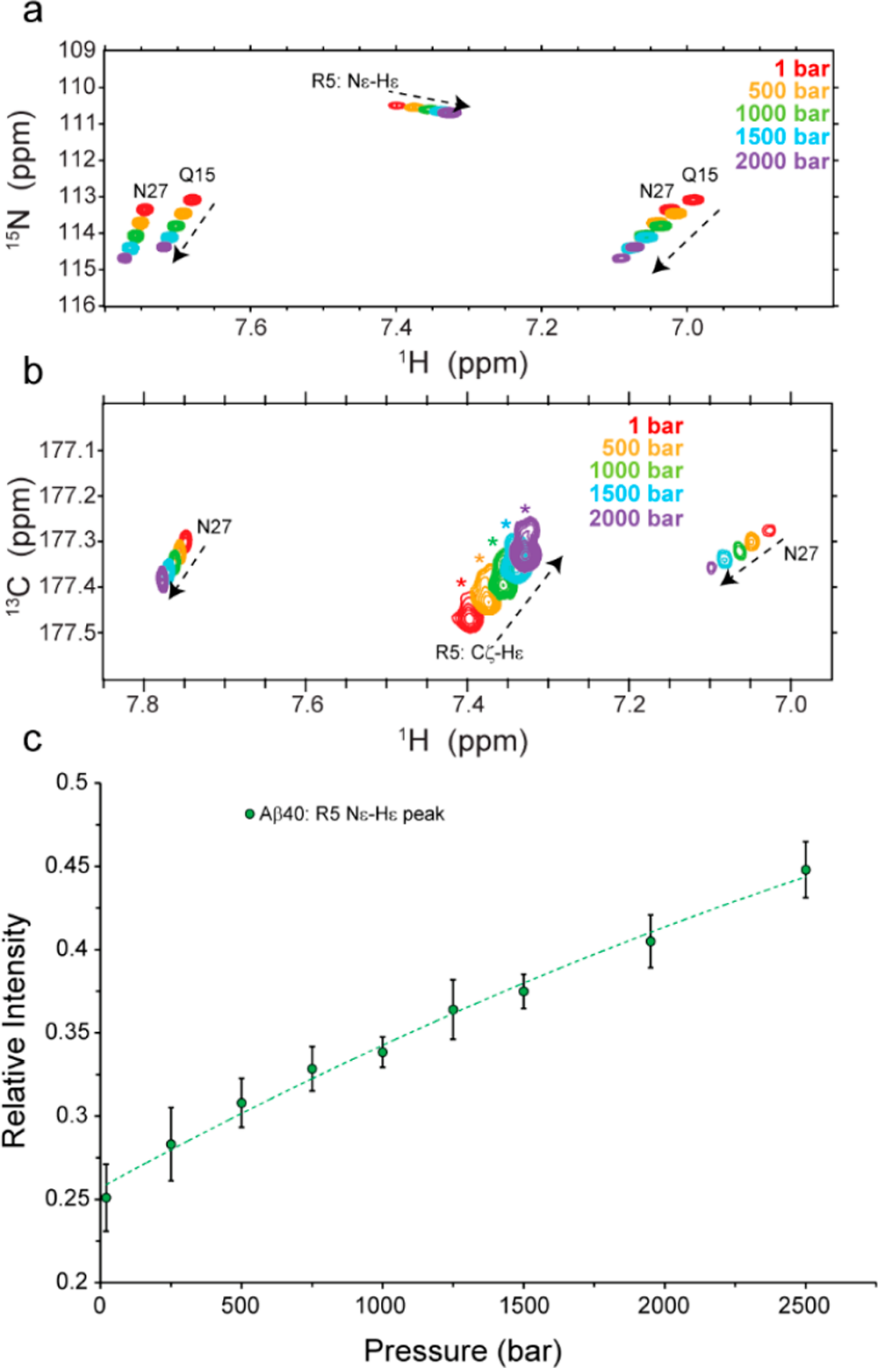
Pressure-induced changes in the Arg5-based salt bridge of Aβ40. (a) Nε-Hε correlation peak in ^15^N,^1^H HSQC and (b) Cζ-Hε correlation peak in the HCO plane of the HNCO spectra. (C) Pressure dependence of the Nε-Hε peak intensity. Note the direction of peak displacements along the proton dimension, which is opposite to that of the backbone (Figure 1a) and also side-chain peaks of Gln15 and Asn27. In panel b, note the pressure-induced enhancement of the satellite peak (highlighted by asterisk). The side-chain peaks of Arg5 are folded in ^15^N and ^13^C dimensions.

To investigate the nature of Arg5-based salt bridges in Aβ40, we utilized a 30 μs long trajectory of Aβ40, one of the longest molecular dynamics (MD) trajectories of Aβ available and validated against an extensive range of experimental data.^29^ The interaction between the guanidinium and carboxylate groups mainly occurs through three different modes: the side-on, end-on, and backside modes (Figure 4a). The side-on and end-on interactions are bidentate configurations predicted by quantum mechanics (QM) calculations to be the lowest energy states, and the backside interaction between the guanidinium group of arginine and the carboxylate groups of aspartate or glutamate is monodentate.^33,34^ In the studied MD trajectory, ∼14% of MD conformers showed a salt bridge between Arg5 and Asp7 side chains, and almost all of them were bidentate in the side-on mode (Table S2). Nearly 10% of conformers contained salt bridges between Arg5 and Glu3 side chains; again, the majority of them were in the side-on mode. There were also ∼6 and 2% of MD conformers with Arg5-Glu11 and Arg5-Asp1 salt bridges. Notably, in a small fraction of MD conformers (∼0.3%), the Arg5 side chain acted as a bridge between Glu3 and Asp7 side chains, making side-on or backside interactions with both of them (Figure 4b). Overall, around one-third of MD conformers contained Arg5-based N-terminal salt bridges. It is worth noting that the MD trajectory contained only a single Aβ40 molecule in the monomeric state; therefore, the intermolecular salt bridges potentially existing in Aβ dimers or small oligomers are not represented here. Subsequently, the effect of N-terminal salt bridges on the overall backbone conformation of Aβ40 in the N-terminal region was investigated. The presence of Glu3-Arg5 or Arg5-Asp7 salt bridges led to a significant reduction in the Cα–Cα distances between residues Glu3 and Ser8 from 1.39 to 1.25 nm and between residues Glu3 and Tyr10 from 1.48 to 1.41 nm. The MD data therefore demonstrate a correlation between Arg5-based salt bridges and less extended N-terminal structures in Aβ.

**Figure 4:**
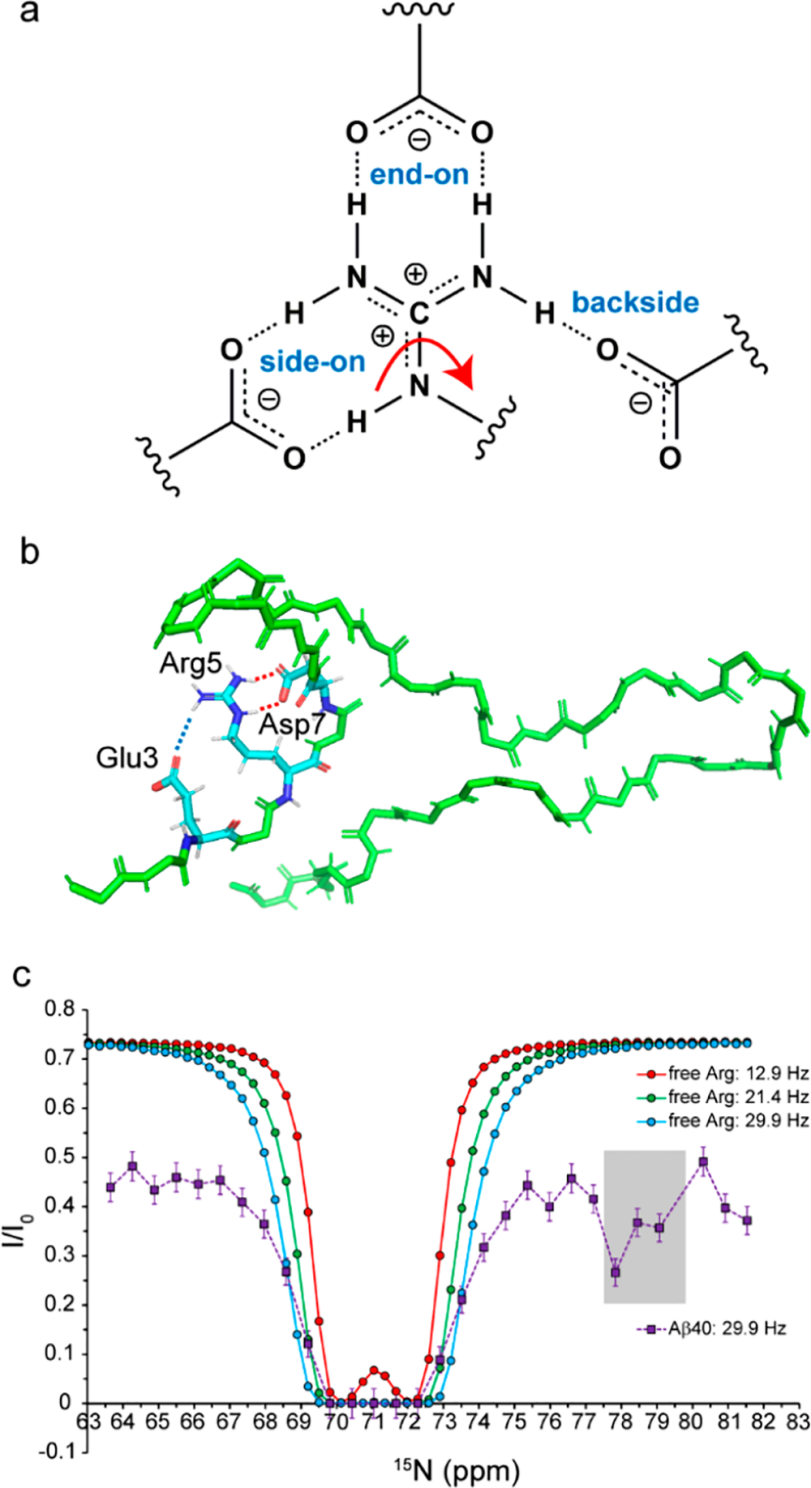
Arginine-based salt bridges in Aβ40. (a) Different modes of the interaction of the guanidinium group of Arg with the carboxylate group of Asp/Glu. (b) Representative conformer of Aβ40 showing two Arg5-based salt bridges: a side-on bridge with Asp7 and a backside bridge with Glu3. (c) MQ-CEST profile of Aβ40s Arg5, showing a minor dip (shaded area) potentially belonging to a salt-bridged Arg5. The profiles of free Arg are shown as a reference.

The arginine guanidinium group undergoes rotational exchange around the Cζ-Nε bond, which leads to the exchange-mediated broadening of the two Nη resonances. A recent ^13^C-detected multiquantum chemical exchange saturation transfer (MQ-CEST) method allows access to Nη chemical shifts through the Cζ-Nε correlation maps that are not affected by the rotational exchange and enables characterization of the rotational dynamics of guanidinium groups in arginine side chains.^35^ The involvement of an arginine side chain in a salt bridge can potentially lead to a change in the chemical shift difference between the two Nη nuclei (Δω in rad/s^–1^) or reduce the rotational dynamics of guanidinium groups (*k*_ex_ in s^–1^). To investigate the potential effect of salt bridge formation on the rotational dynamics of the guanidinium group in Arg5 of Aβ40, we measured the MQ-CEST of Aβ40 and compared it with that of the free arginine as a reference (Figure 4c). In free arginine, where the guanidinium rotation is largely unrestricted, the MQ-CEST profile showed a CEST dip at ca. 71 ppm and provided a *k*_ex_ of 356 ± 8 s^–1^ and Δω of 1214 ± 6 rad/s^–1^ at 274 K, indicating a slow-to-intermediate chemical exchange regime for its two Nη nuclei. On the contrary, in Aβ40, in addition to a similar CEST dip at ca.71 ppm, a minor dip was detected at ca. 77–79 ppm. The observation of this minor dip provides further support for the (partial) presence of Arg5-based salt bridges in Aβ, as the Nη chemical shifts of the minor species (with the salt bridge) with respect to the major species (without the salt bridge) are in line with the DFT-based prediction of the Nη chemical shifts of a model peptide with/without salt bridges (Figure S8, Table S1). The presence of the minor dip did not, however, allow a two-site symmetrical exchange-based analysis of the MQ-CEST profile; therefore, no reliable *k*_ex_ or Δω could be obtained for the Arg5 of Aβ40.

The phosphorylation of Aβ at Ser8 enhances its aggregation and alters the morphology, structure, dynamics, and stability of Aβ fibrils.^16,36−40^ To explore the possible mechanism(s) underlying the aggregation-promoting effect of Ser8 phosphorylation, we investigated how the introduction of the negatively charged phosphoserine 8 influences the network of electrostatic interactions, including salt bridges, in the N-terminal region of Aβ. To this end, we performed DFT calculations of the Aβ structures in the presence or absence of serine 8 phosphorylation. Two sets of Aβ conformers containing (test group) or lacking (control group) the Arg5-based salt bridges were selected from the MD trajectory (Table 1). To each Aβ conformer was added a phosphate group at the Ser8 side chain, and the energy difference between the phosphorylated and nonphosphorylated Aβ was calculated. (See the Supplementary Methods and Figure S6.) When compared with the control group, the test group containing Arg5-based salt bridges was relatively destabilized upon Ser8 phosphorylation, especially when both the Arg5-Asp7 and Arg5-Glu3 salt bridges were present (Table 1). Notably, the overall energetic effect of phosphorylation is determined by a complex network of interactions, including the direct interaction between Arg5 and phosphoserine side chains. Therefore, without further investigation and disentangling of separate contributions, the energetic effect of phosphorylation cannot be simply attributed to the presence or absence of a single salt bridge.

**Table 1:** Density Functional Theory (DFT)-based Energy Calculation of Representative N-Terminal Aβ Conformers Containing or Lacking Arg5-based Salt Bridges in the Ser8-Phosphorylated and Nonphosphorylated Forms

	number of conformers	R5-E3 salt bridge	R5-D7 salt bridge	Δ*E*_pS8-np_ (hartrees)^a^	ΔΔ*E* (hartrees)^b^	ΔΔ*E* (kJ/mol)^b^
test group	7	present	present	–567.268 ± 0.065	0.035	90.96
	8	absent	present	–567.291 ± 0.026	0.013	32.84
control group	12	absent	absent	–567.303 ± 0.023		

a Energy difference between the Ser8-phosphorylated and nonphosphorylated conformers of Aβ in hartrees.

b Difference in the Ser8-phosphorylation-induced stabilization energy between test and control groups in hartrees and kJ/mol. The positive value means relative destabilization of test conformers when compared with the control group.

The DFT results suggest that the Ser8 phosphorylation of Aβ induces a shift in the conformational ensemble of Aβ toward conformers in which the Arg5-based salt bridges are partially disrupted. Consequently, the Ser-8 phosphorylated Aβ is expected to be relatively extended in the N-terminal region. This is consistent with the Hα chemical shift changes induced upon Ser8 phosphorylation, as previously reported.^36^ The Ser8-phosphorylation-induced disruption of the Arg5-based salt bridges and its resultant increase in the mobility of the Arg5 side chain is supported by the (partial) loss of Arg5’s Hβ resonance dispersion in Ser8-phosphorylated Aβ (Figure S7a,b). Further support for the lower population of Arg5-based salt bridges within the pS8-Aβ40 ensemble is provided by the smaller negative pressure coefficient of Arg5’s Hε chemical shifts in pS8-Aβ40 compared with the nonphosphorylated Aβ40 (Figure S7c).

It has been suggested that the N-terminal region of Aβ plays an important role in controlling the aggregation pathway of Aβ, even if it remains largely unstructured in the final Aβ fibrils.^19,20,37^ Our combined high-pressure NMR and MD simulation data point to the presence of N-terminal salt bridges in Aβ favoring relatively rigid compact structures in the N-terminal region. The presence of a relatively compact (sub)ensemble of Aβ monomers in rapid exchange with largely unstructured Aβ monomers has been previously shown.^24^ It is expected that the partial disruption of the N-terminal salt bridges caused by Ser8-phosphorylation, as proposed by our DFT calculations, induces a relatively mobile extended structure in the N-terminal region of Aβ and enables the long-range interactions between the N-terminus and the rest of amyloid core observed in pSer8-Aβ fibrils.^37,38^ Furthermore, it is notable that the brain-derived Aβ fibrils contain Arg5-Glu3 salt bridges between adjacent protofibrils, suggesting that the Arg5-based salt bridges may play a role in the higher order assembly of Aβ aggregates as well.^22^ Thus our results put forward the hypothesis that the network of electrostatic interactions in the N-terminal region of Aβ may act as a regulatory switch in Aβ aggregation. Accordingly, modulation of N-terminal electrostatic interactions through charge-altering mutations (such as D7N) or modifications (such as phosphorylation or N-terminal cleavage), pH, metal ion binding, or proximity to phospholipid membranes can alter the balance between local and long-range interactions and control the kinetics of Aβ aggregation and the morphology and structure of Aβ aggregates. However, the formation of long-range interactions involving N-terminal residues may not occur during early Aβ oligomerization, as suggested by a pressure-jump NMR study.^41^

To summarize, we have shown that the structural dynamics of Aβ are significantly influenced by pressure increase, largely in favor of more extended structures with higher backbone dynamics and because of the disruption of local electrostatic interactions. In particular, the high-pressure NMR data provided experimental support for the transient formation of Arg5-based salt bridges in Aβ in accord with the MD simulation and multiquantum CEST data. Using a combination of MD simulation and DFT calculations, we demonstrated that the aggregation-promoting phosphorylation of Ser8 can alter the network of electrostatic interactions and thus induce conformational changes in the N-terminus of Aβ. The induced structures are proposed to represent the early aggregation-competent conformers of Aβ and can be used as potential targets in developing drugs against AD.

## Supporting Information Available

Description of NMR experiments, MD simulation and MD-based analysis of salt bridges, DFT calculations, Supplementary Figures S1–S8, and Tables S1 and S2 (PDF)

## Notes

All DFT materials are available upon request.
