# Supplementary material for: Combined High-Pressure and Multiquantum NMR and Molecular Simulation Propose a Role for N-Terminal Salt Bridges in Amyloid-Beta
Source: J Phys Chem Lett. 2021 Oct 7;12(40):9933–9. doi: 10.1021/acs.jpclett.1c02595 (PMC8521524; doi:10.1021/acs.jpclett.1c02595)
Supplement: Supplementary file 1 — jz1c02595_si_001.pdf [file jz1c02595_si_001.pdf]

Combined High-Pressure and Multiquantum NMR and Molecular Simulation Proposes a Role for N-terminal Salt Bridges in Amyloid-Beta

Sahithya Phani Babu Vemulapalli[†,§], Stefan Becker[†], Christian Griesinger[†], Nasrollah Rezaei-Ghaleh[†,‡,#*]

† Department of NMR-based Structural Biology, Max Planck Institute for Biophysical Chemistry, Göttingen, Germany
§ Institute for Chemistry and Biology of the Marine Environment, University of Oldenburg, Oldenburg, Germany
‡ Department of Neurology, University Medical Center Göttingen, Göttingen, Germany
# Institute for Physical Biology, Heinrich Heine University, Düsseldorf, Germany

*Correspondence to: Dr. N. Rezaei-Ghaleh; Nasrollah.Rezaie.Ghaleh@hhu.de

Supplementary Methods

Materials

The synthetic non-phosphorylated and Ser8-phosphorylated Aβ1-40 (Aβ40) peptides were purchased from Peptide Special Laboratory (PSL, Heidelberg, Germany). Uniformly ^{15}N,^{13}C-labelled Aβ40 and Aβ42 were produced recombinantly, as described before.[1] To dissolve the pre-formed peptide aggregates, the lyophilized Aβ40 and Aβ42 peptides were solubilized in 20 and 50 mM NaOH at a concentration of 2 and 1 mg/mL, respectively, and stored at -80 °C until use.[2]

High-pressure NMR experiments

All high-pressure NMR experiments were measured on Avance III HD 700 MHz NMR spectrometer equipped with a 5 mm TCI cryo probe (Bruker, Germany). NMR samples contained 0.3 mg/mL (ca. 70 μM) Aβ40 or 0.15 mg/mL (ca. 33 μM) Aβ42, both buffered with 20 mM sodium phosphate at pH 7.4, in which the Aβ40 and Aβ42 concentrations were well below the threshold peptide concentration for oligomerization at ambient pressure.[3] The NMR samples contained 4,4-dimethyl-4-silapentane-1-sulfonic acid (DSS) for chemical shift referencing at different pressure levels and 10% D_2O for the field frequency locking. Pressure-resistant ceramic NMR tubes were used for all the experiments and pressure levels ranging from 1 to 2000 bar were applied to the NMR samples (Daedalus Innovations LLC, PA). Temperature was controlled to ±0.05 K using the Bruker VT unit. Standard ^{15}N,^{1}H HSQC, ^{13}C,^{1}H HSQC and HNCO spectra were measured at 278 K and various pressure levels ranging from 1 to 2000 bar. The high-pressure ^{1}H,^{1}H TOCSY spectra were measured using samples containing 0.4 mg/mL (ca. 95 μM) non-

phosphorylated or Ser-8 phosphorylated (synthetic) Aβ40 in 25 mM HEPES (pH 7.2), a ceramic NMR tube resistant to pressures up to 1000 bar and mixing time of 60 ms. Chemical shift referencing of $^{1}H,^{1}H$ TOCSY spectra at each pressure level was done using the DSS signal, as mentioned above. NMR spectra were processed and analysed using NMRPipe[4] and Sparky 3.114 (T.D. Goddard and D.G. Kneller, http://www.cgl.ucsf.edu/home/sparky).

Backbone and side-chain resonance assignments were obtained through standard sequential assignment strategy at 1 bar, supported by our previous reports,[1] and then transferred to the spectra obtained at other pressure levels by gradually incrementing the pressure (at steps of 250 bar). The backbone chemical shifts Cα, Cβ, CO, N, HN and Hα were used to predict distinct protein structural motifs and calculate random coil index (RCI)-based squared order parameters (S^2), after automated correction for chemical shift offsets.[5-6]

Pressure coefficients of backbone amide ^{1}H and ^{15}N chemical shifts were calculated as described in [7]. First, the random-coil chemical shifts of the amide ^{1}H and ^{15}N nuclei of each amino acid X at each given pressure (*P*) were calculated from the known random-coil chemical shifts in the model peptide Ac-Gly-Gly-X-Ala-NH2 at ambient pressure (P_0) and their pressure dependence, as determined by Koehler *et al.*[8]. Then, the corrected ^{1}H and ^{15}N shifts (δ*) were fitted to the following second order Taylor expansion:

$$\boldsymbol{\delta^{*}_{p} = \delta^{*}_{0} + B^{*}_{1}(P - P_{0}) + \frac{1}{2}\, B^{*}_{2}\, (P - P_{0})^{2}} \qquad \text{Eq. S1}$$

and first- (B_1) and second-order (B_2) pressure coefficients were determined separately for amide ^{1}H and ^{15}N nuclei of different residues. This approach enables correcting for the direct effect of pressure on the chemical shifts of each amino acid, especially in the titratable residues such as histidine in which the pKa of side-chains are pressure-dependent.

Multi-quantum Chemical Exchange Saturation Transfer (MQ-CEST) NMR experiments

The NMR samples contained 100 mM free [$^{13}C_6$,$^{15}N_4$]-L-arginine (25 mM HEPES, pH 5.1, 10% D_2O) or ca. 80 μM ^{13}C,^{15}N-labeled Aβ40 (25 mM HEPES, 50 mM NaCl, pH 6.4, 10% D_2O). The MQ-CEST experiments were measured on a Bruker Avance Neo 800 MHz NMR spectrometer equipped with a 3 mm TCI cryo probe at 274 K, following the NMR pulse sequence introduced by Karunanithy *et al* [9]. Briefly, this is a ^{13}C-detected NMR experiment starting with the steady-state longitudinal magnetization on the $^{1}H^{\varepsilon}$ spins of arginine sidechains and correlating the chemical shifts of the $^{13}C^{\zeta}$ spins of arginine side chains in the direct dimension to the chemical shifts of their scalar coupled $^{15}N^{\varepsilon}$ spins in the indirect dimension. At a specific point during the sequence a three-spin order density element proportional to $4Cz^{\zeta}Nz^{\eta}Nz^{\eta}$ is generated, which is then subjected to a weak ^{15}N B_1 field during the CEST period, where the ^{15}N carrier frequency is varied over a range covering the chemical shifts of the two N^{η} spins of arginine sidechains. As a result, the CEST

intensities are obtained from the intensity of the N^{ε}-C^{ζ} correlation peaks as a function of the ^{15}N carrier offset. In the present study, the ^{15}N CEST elements were 250 ms long, during which the ^{15}N B_1 fields of 12.9, 21.4 and 29.9 Hz for the free arginine sample or 29.9 Hz for the Aβ40 sample were applied. For the reference free arginine sample, 61 evenly spaced ^{15}N carrier offsets between 63.0 and 81.5 ppm at 25 Hz (ca. 0.31 ppm) intervals were used during the ^{15}N CEST element. For the much lower concentrated Aβ40 sample 30 evenly spaced ^{15}N offsets between 63.6 and 81.5 ppm at 50 Hz (ca. 0.62 ppm) intervals were used. At each ^{15}N B_1 field value a reference spectrum was recorded without the CEST element (T_{CEST} = 0), but including the heat-compensating element of identical duration before recycle delay (d_1), during which the same ^{15}N B_1 field was applied far off-resonance (at 232 ppm). The ^{15}N offset-dependent CEST intensities were presented as the intensity ratios (I/I_0) with respect to the N^{ε}-C^{ζ} peak intensity in the reference spectrum (I_0). The ^{15}N B_1 field strengths were calibrated as described in reference [10]. It is worth mentioning that the sensitivity of MQ-CEST experiment is highly dependent on water-Hε exchange rate, therefore the pH of sample had to be reduced (from 7.4 of other experiments) to 6.4 for the sake of sensitivity.

Two-dimensional homonuclear NMR experiments

The ^{1}H,^{1}H TOCSY experiments were performed on a Bruker (Germany) Avance 800 MHz spectrometer equipped with a cryogenic probe, as in [11]. Briefly, the NMR samples contained 0.4 mg/mL Aβ in 20 mM sodium phosphate buffer, pH 7.2. The NMR measurements were performed at 278 K. The time domain data contained 2,048 and 600 complex data points in t2 and t1, respectively. The TOCSY mixing time was 60 ms.

Molecular Dynamics (MD) simulation

The MD trajectory of ref. [12] was analysed. There, the ca. 30-μs long MD simulation of Aβ40 was performed using the a99SB-*disp* force field with the optimized TIP4P-D water model at 1 bar and 300 K. The 30,000 MD frames saved at 1 ns intervals were analysed in this study. The salt bridges were identified using a cut-off of 4 Å between N-O atom pairs of basic (Arg and Lys) and acidic (Asp and Glu) residues.[13]

Density Functional Theory (DFT) calculations

All DFT energy calculations of a 9-residue (^{2}AEFRHDSGY^{10}) N-terminal sequence of Aβ40 were carried out using Gaussian 09.[14] The Aβ40 conformers obtained from the MD trajectory were used for DFT calculation of energy without further geometry optimization. N- and C-termini of a 9-residue (^{2}AEFRHDSGY^{10}) fragment of Aβ40 were capped with methyl groups. The energy calculations were performed at DFT level using hybrid meta exchange-correlation functional M06-2X[15-17] and Def2TZVPP[18-19] basis set. Integral equation formalism polarizable continuum model (IEFPCM)[20] was employed with water as a solvent. For each conformer two energy

calculations were performed with and without the presence of doubly negative charged phosphate group of serine-8 residue.

A model pentapeptide Glu-Gly-Arg-Gly-Asp conformers with (Figure S8a) and without (Figure S8b) salt bridge between Arg and Glu/Asp side chains were used for the NMR shielding tensor calculation of the guanidinium group of Arg using Gaussian 09. Geometry optimization of both the conformers was carried out at DFT/B3LYP[21-24]/6-31G(d)[25] level of theory and by employing IEFPCM solvent model with water as a solvent (for Cartesian coordinates of the energy-minimized model peptide in two conformations, please see below). The energy minimized conformers were used as input geometries for the NMR shielding tensor calculations using gauge-independent atomic orbital (GIAO)[26] method at DFT/mPW1PW91[27-28]/6-311+G(2d,p) level of theory and by employing IEFPCM solvent model with water as a solvent. The calculated ^{1}H and ^{13}C nuclear shielding tensors were converted into chemical shifts using the scaling factors obtained from the CHESHIRE (chemical shift repository)[29] web site. The scaled chemical shifts were derived by substituting the scaling factors for ^{1}H (slope: -1.0651; intercept: 31.8547) and ^{13}C (slope: -1.0275; intercept: 185.7787) into the following equation;

$$\delta = \frac{intercept - \sigma}{-slope}$$

where δ is the scaled chemical shift value relative to TMS and σ is the computed isotropic value. The computed ^{15}N nuclear shielding tensors were converted into chemical shifts relative to the calculated ^{15}N chemical shift of ammonia.

Supplementary Figures

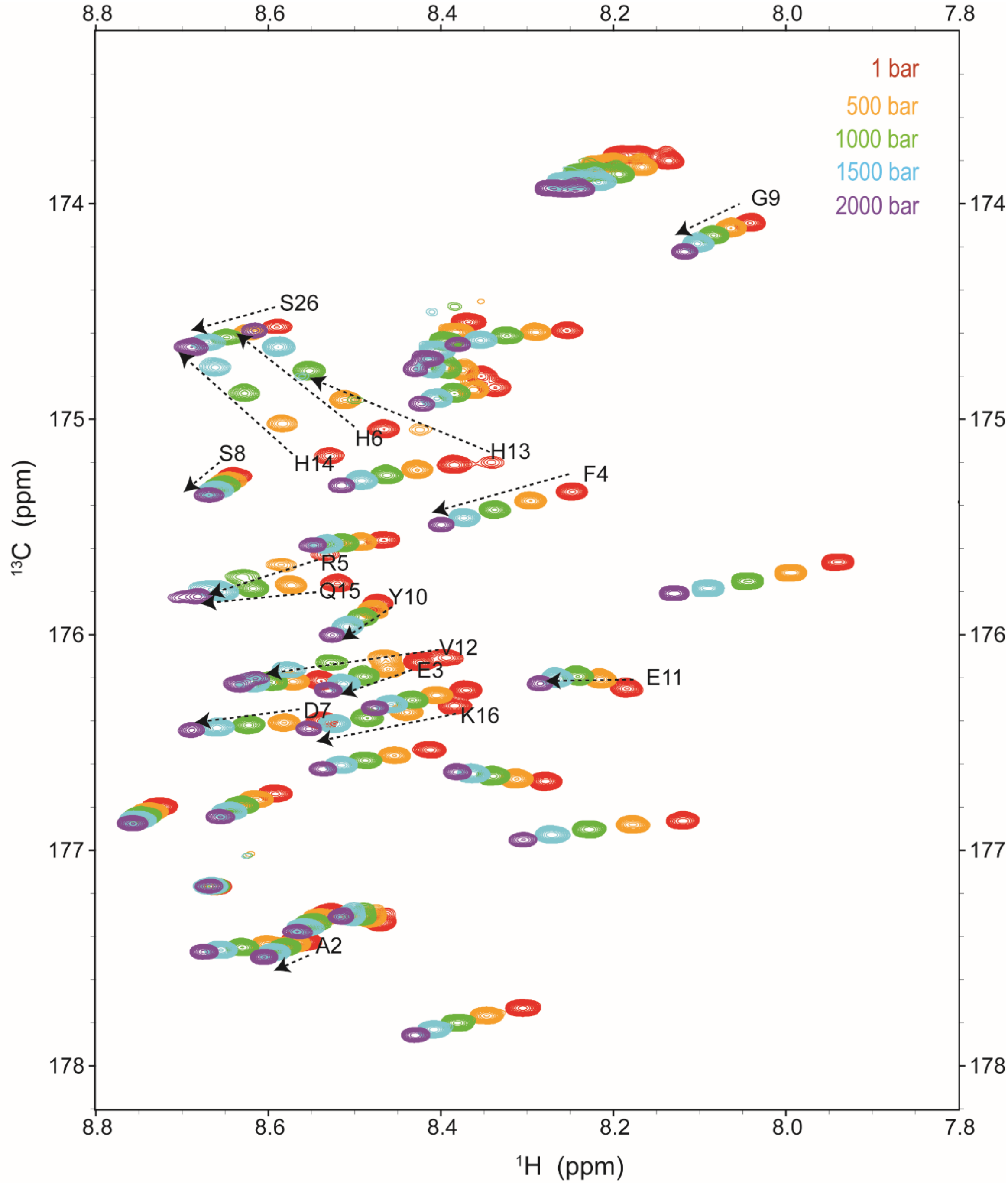

Figure S1. Pressure-dependence of NMR spectra of Aβ40. The HCO plane of HNCO spectra obtained at 1, 500, 1000, 1500 and 2000 bar are shown. Note the direction of peak displacement, which are highlighted by dotted arrows.

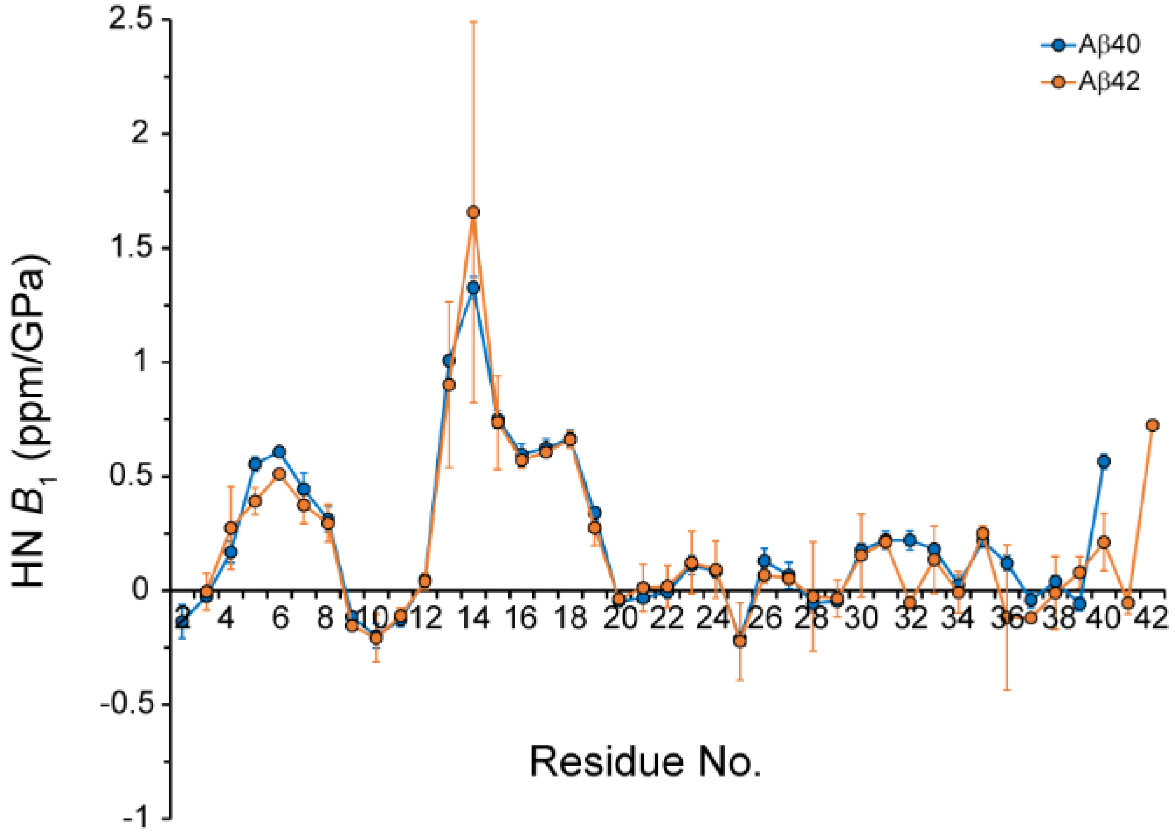

Figure S2. The first-order (B_1) pressure coefficients of the backbone amide protons over the sequence of Aβ40 (blue) and Aβ42 (orange) peptides. The B_1 coefficients were calculated after correction for the pressure effects on the chemical shifts of random coil model peptides. Two regions, Arg5-Ser8 and His13-Phe19 show relatively large positive B_1 values.

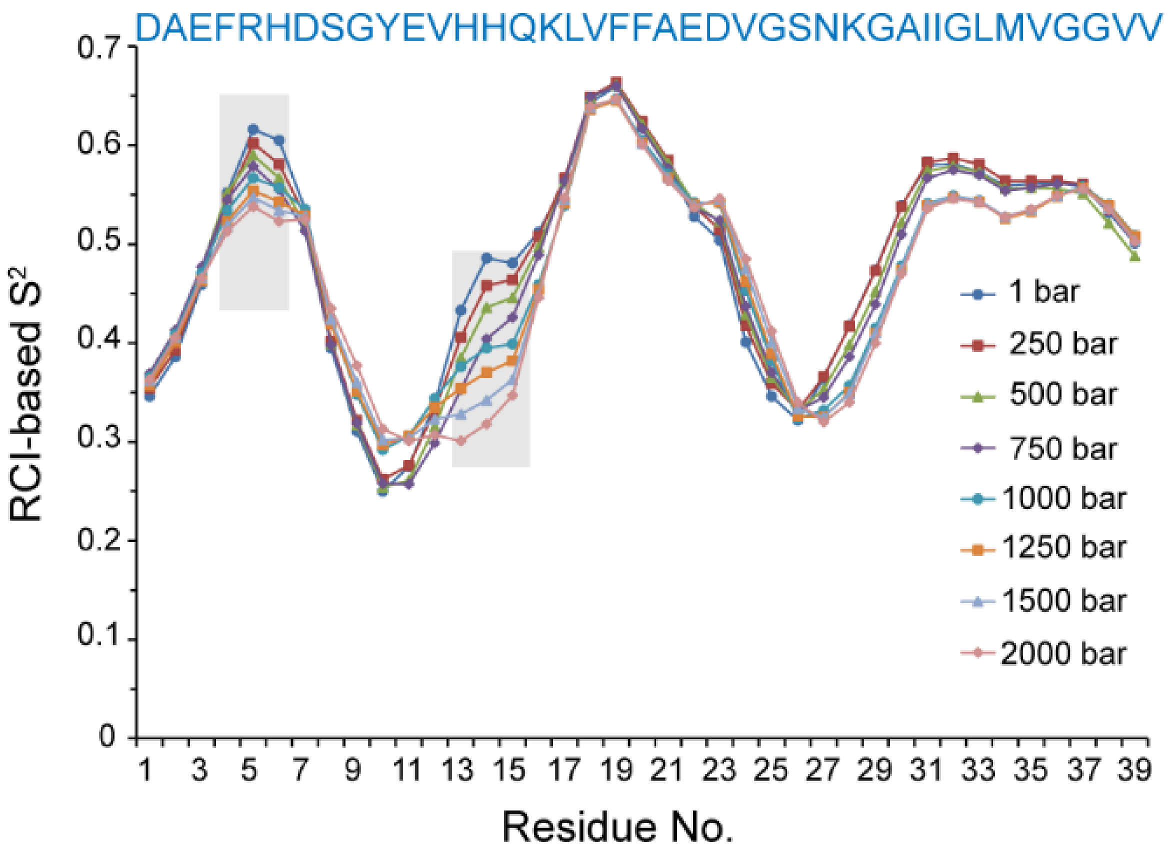

Figure S3. Residue-specific squared order parameters (S^2) of Aβ40 in dependence of pressure, based on the Random Coil Index (RCI) values. Residues R5-H6 and H13-K16 exhibit bigger probability of strand formation and smaller order parameters, i.e. larger mobility, at higher pressures (shaded boxes).

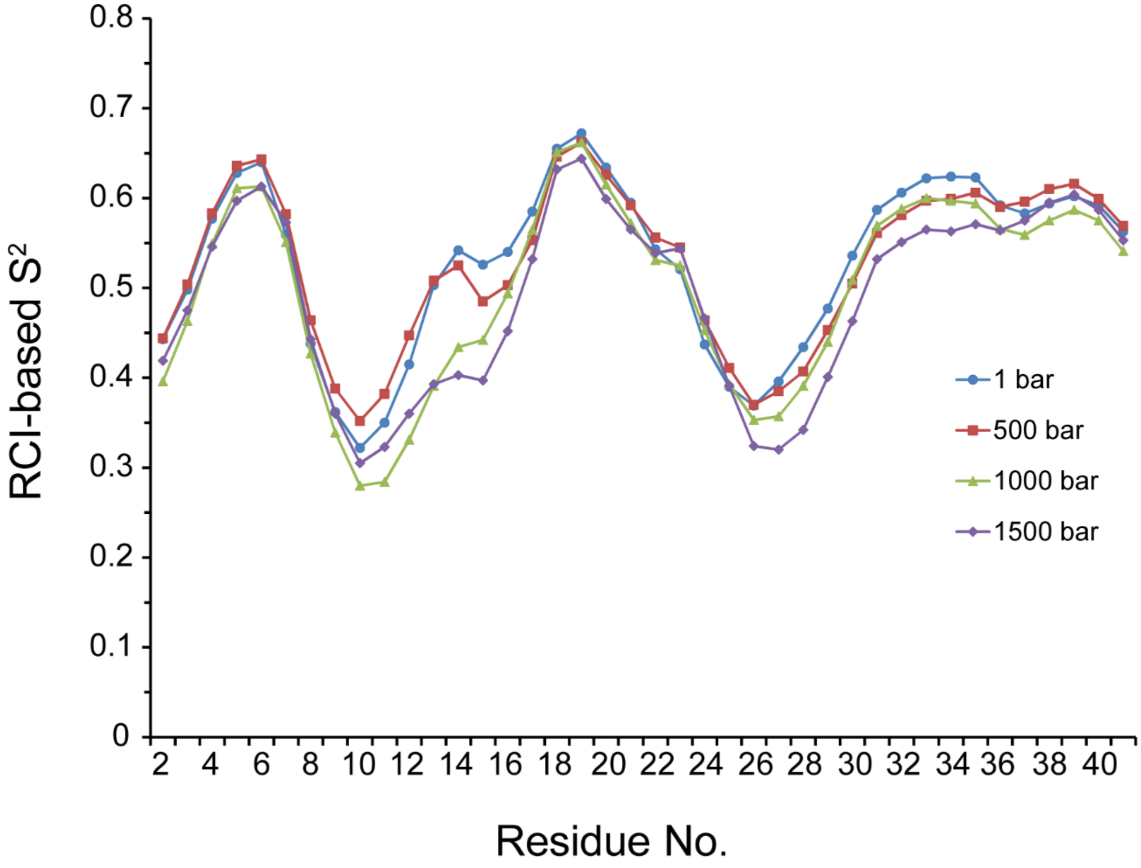

Figure S4. Residue-specific squared order parameters (S^2) of Aβ42 in dependence of pressure, based on the Random Coil Index (RCI) values derived from backbone (CO, Cα, Cβ, N, HN, Hα) chemical shifts. The backbone mobility is generally increased at high pressure.

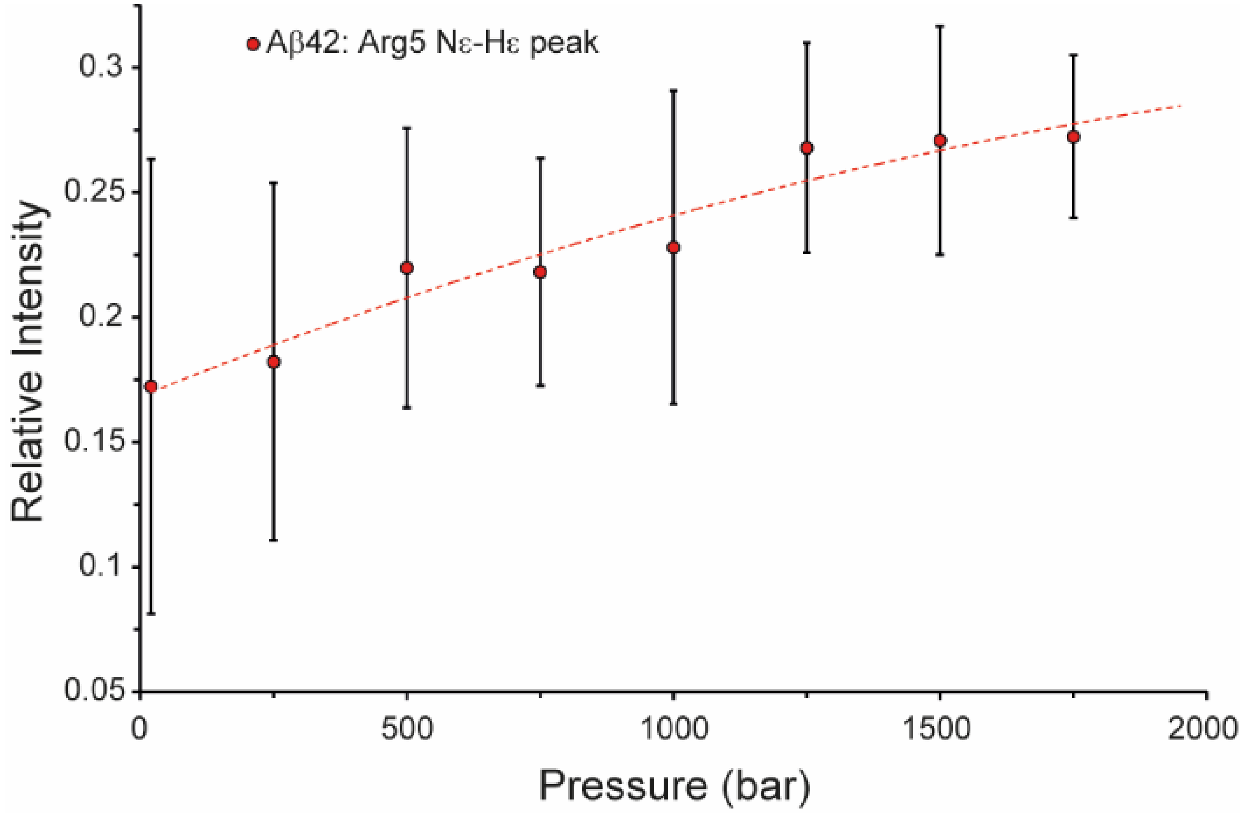

Figure S5. Pressure-dependence of the peak intensity of Arg5 side-chain in $^{15}N,^{1}H$ HSQC spectrum of Aβ42 measured at 278 K. The peak intensities are normalized with the average peak intensity of backbone peaks. The errors were determined on the basis of signal-to-noise ratio.

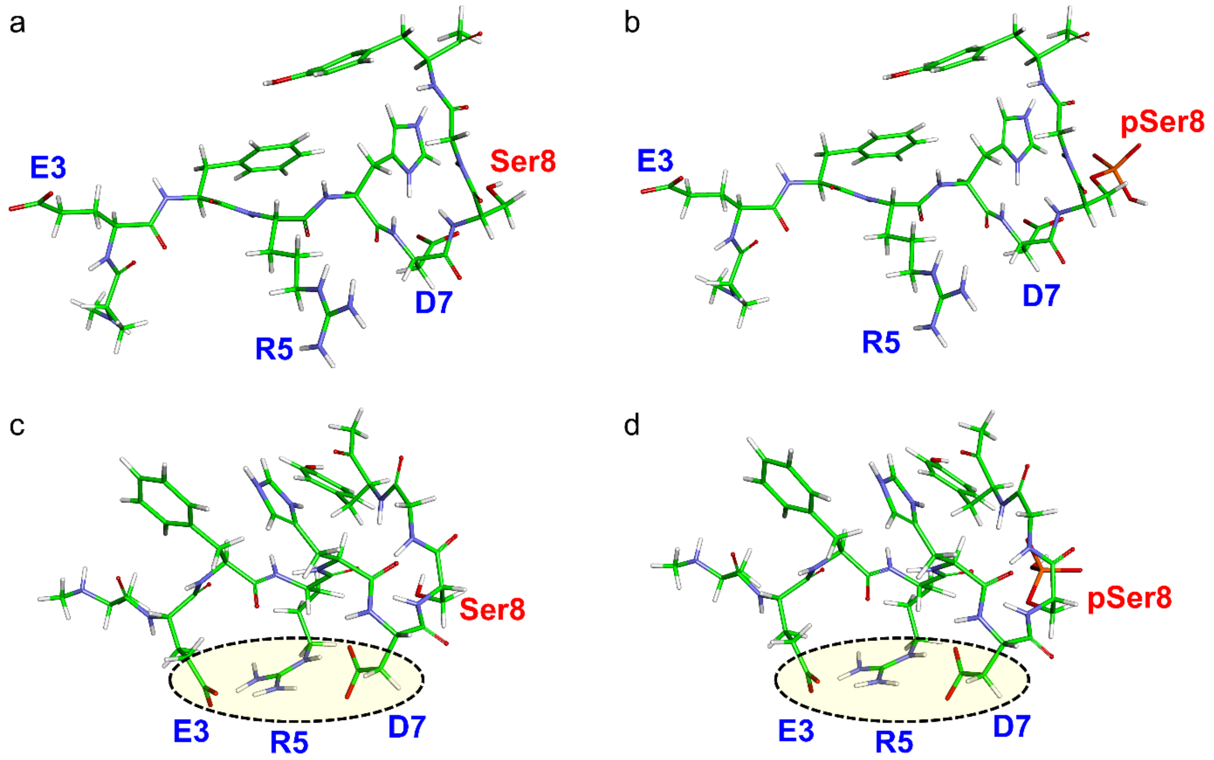

Figure S6. Representative structures of a 9-residue (^{2}AEFRHDSGY^{10}) N-terminal sequence of Aβ40 without and with phosphorylated-Ser8 used for the DFT energy calculations: (a,b) control group and (c,d) test group. Dotted ellipse highlights the presence of R5-E3 and R5-D7 salt bridges in the test group structures.

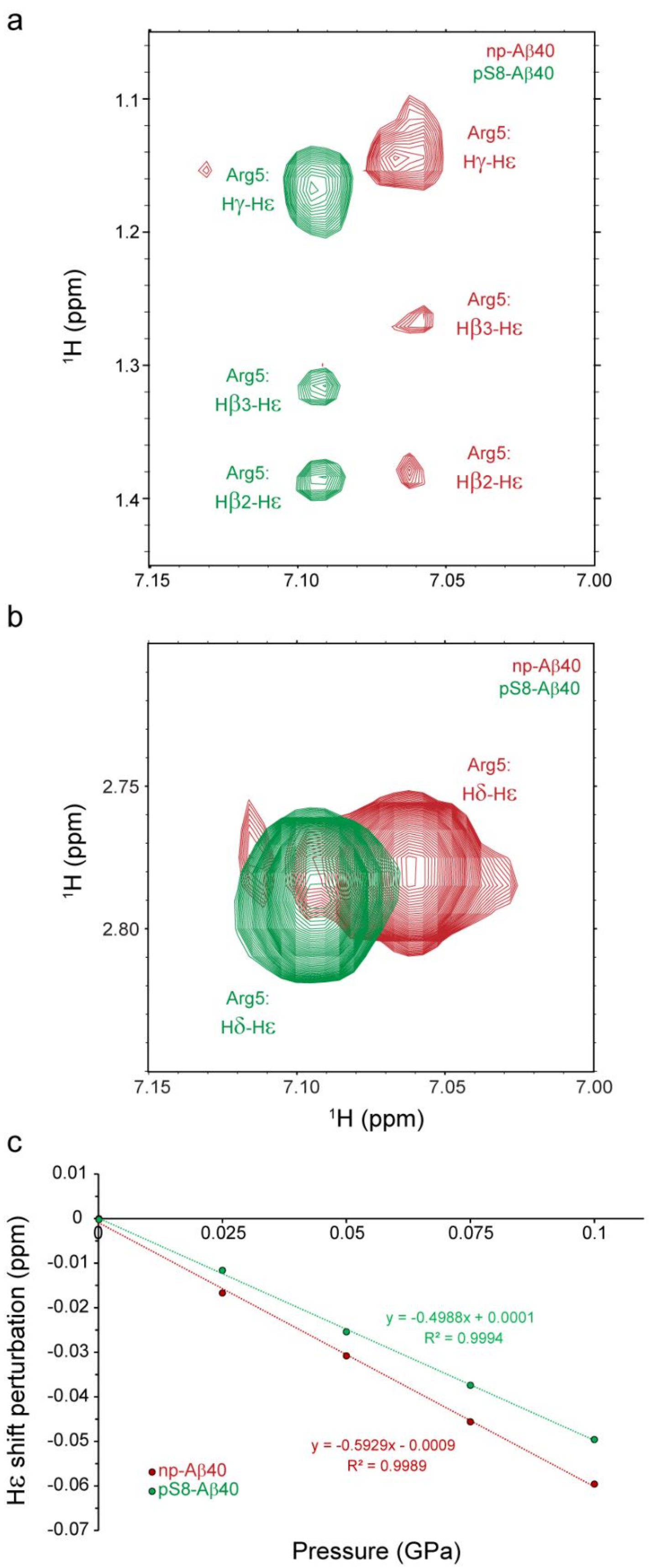
a
np-Aβ40
pS8-Aβ40
Arg5: Hγ-Hε
Arg5: Hγ-Hε
Arg5: Hβ3-Hε
Arg5: Hβ3-Hε
Arg5: Hβ2-Hε
Arg5: Hβ2-Hε
1H (ppm)
1.1
1.2
1.3
1.4
7.15
7.10
7.05
7.00
b
np-Aβ40
pS8-Aβ40
Arg5: Hδ-Hε
Arg5: Hδ-Hε
1H (ppm)
2.75
2.80
7.15
7.10
7.05
7.00
1H (ppm)
c
Hε shift perturbation (ppm)
0.01
0
-0.01
-0.02
-0.03
-0.04
-0.05
-0.06
-0.07
0
0.025
0.05
0.075
0.1
y = -0.4988x + 0.0001
R² = 0.9994
y = -0.5929x - 0.0009
R² = 0.9989
np-Aβ40
pS8-Aβ40
Pressure (GPa)

Figure S7. TOCSY-based correlation of the Hε resonances of Arg5's side chain with the Hγ and Hβ (panel a) and Hδ (panel b) resonances, in non-phosphorylated (red) and Ser8-phosphorylated Aβ40 (green). The Ser8 phosphorylation leads to a partial loss in the dispersion of Hβ resonances, consistent with the enhanced dynamics of Arg5 side chain induced by the disruption of Arg5-based salt bridges. In panels (a) and (b) the chemical shifts have been referenced with respect to water signal (4.700 ppm). The pressure-induced perturbation of Hε chemical shifts are shown in panel (c), with Ser8-phosphorylated Aβ exhibiting a significantly lower negative pressure coefficient than the non-phosphorylated Aβ peptide (-0.4988 ppm/GPa with 95% CI of -0.5222 to -0.4754 *vs* -0.5929 ppm/GPa with 95% CI of -0.6285 to -0.5573).

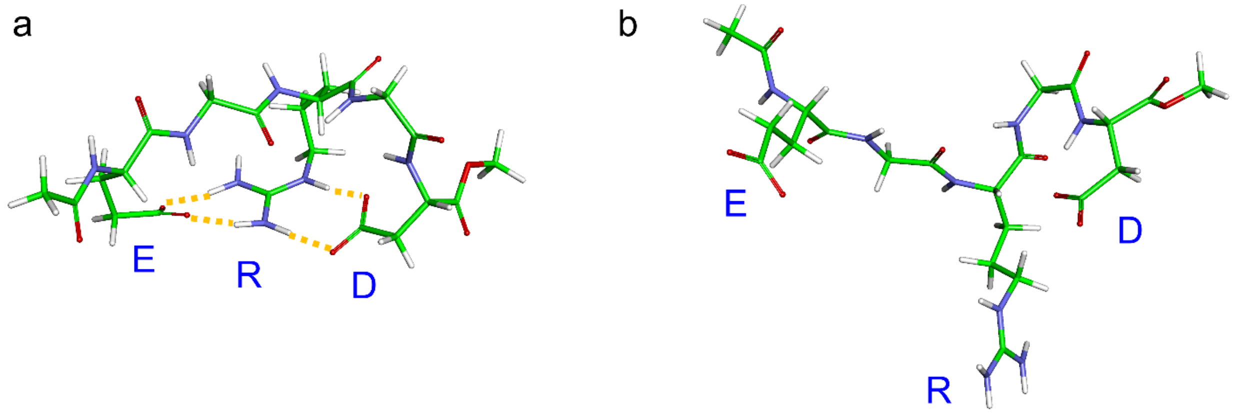

Figure S8: The model pentapeptide Glu-Gly-Arg-Gly-Asp (EGRGD) conformers with (a) and without (b) salt bridge between arginine and glutamate/aspartate side chains, used for computing the NMR shielding tensors of guanidinium group of Arg in dependence of salt bridge formation/disruption. Yellow dotted lines indicate the salt bridge interaction between the guanidinium group of Arg and carboxylate groups of Glu/Asp.

Cartesian coordinates of the energy minimized conformation of model pentapeptide Glu-Gly-Arg-Gly-Asp with the salt bridge between Arg and Glu/Asp, used for the Arg side chain nuclear shielding tensor calculations.

C	5.849	28.425	17.198
O	5.267	28.494	18.292
N	5.593	27.319	16.388
C	4.725	26.143	16.677
C	5.223	25.001	17.562
O	6.364	24.651	17.639
C	4.154	25.642	15.372
C	2.81	24.89	15.511
C	2.422	24.087	14.312
O	1.411	23.33	14.456
O	3.024	24.088	13.238
H	5.963	27.354	15.449
H	3.888	26.674	17.13
H	4.159	26.39	14.579
H	4.895	24.957	14.96
H	2.871	24.167	16.324
H	1.953	25.523	15.739
N	4.343	24.451	18.395
C	4.579	23.284	19.258
C	4.607	22.085	18.292
O	3.621	21.815	17.579
H	3.387	24.771	18.455
H	5.462	23.402	19.885
N	5.714	21.367	18.306
C	5.846	20.058	17.627
C	5.269	18.874	18.418
O	5.894	17.81	18.511
C	7.269	19.854	17.045
C	7.596	20.917	15.952
C	7.29	20.388	14.516
N	5.79	20.391	14.286
C	4.965	21.41	13.892
N	5.36	22.54	13.517
N	3.684	21.256	13.798
H	6.465	21.699	18.894
H	5.261	19.946	16.714
H	7.931	20.04	17.89
H	7.494	18.839	16.717
H	7.095	21.861	16.167
H	8.684	20.947	16.008
H	7.796	20.986	13.758
H	7.642	19.37	14.349
H	5.279	19.593	14.636
H	6.254	22.844	13.874
H	4.731	23.278	13.231

H	3.293	20.342	13.973
H	3.114	22.086	13.718
N	4.099	19.167	19.05
C	3.284	18.082	19.76
C	3.225	16.74	18.921
O	3.434	15.708	19.517
H	3.691	20.09	19.028
H	3.904	17.856	20.628
N	3.056	16.863	17.561
C	2.965	15.759	16.553
C	4.165	14.838	16.483
O	4.148	13.88	15.76
C	2.653	16.489	15.166
C	3.579	17.578	14.714
O	4.527	18.084	15.394
O	3.319	18.098	13.598
H	3.096	17.826	17.259
H	2.139	15.117	16.858
H	2.517	15.74	14.386
H	1.668	16.95	15.235
C	6.41039	14.26987	17.48548
H	5.99485	13.38331	17.91705
H	6.9918	14.00934	16.62584
H	7.03528	14.75737	18.20433
O	5.324	15.176	17.086
C	6.73837	29.5088	16.56081
H	6.24928	29.90942	15.69756
H	6.9093	30.292	17.26953
H	7.67446	29.078	16.27262
H	2.38599	18.46182	20.2007
H	3.75754	23.22543	19.94114

Cartesian coordinates of the energy minimized conformation of model pentapeptide Glu-Gly-Arg-Gly-Asp without the salt bridge between Arg and Glu/Asp, used for the Arg side chain nuclear shielding tensor calculations.

C	-6.89896	-2.16425	-1.06715
O	-6.79359	-1.61133	-2.16818
N	-6.18678	-1.77352	0.01524
C	-5.19677	-0.71104	-0.03315
C	-4.27819	-0.91927	1.18404
O	-4.64515	-1.58404	2.16129
C	-5.84017	0.71738	-0.02314
C	-6.77768	1.01582	-1.21162
C	-7.42503	2.40719	-1.1594
O	-6.64416	3.40347	-1.33863
O	-8.65898	2.48185	-0.92629
H	-6.25921	-2.25732	0.90308
H	-4.61053	-0.81576	-0.94989

H	-6.42153	0.80273	0.90002
H	-5.05972	1.48699	0.03267
H	-6.19475	0.93018	-2.13627
H	-7.56972	0.26064	-1.23267
N	-3.08393	-0.29997	1.10123
C	-2.14409	-0.29679	2.20138
C	-0.7059	-0.27261	1.6844
O	-0.39161	-0.7787	0.60069
H	-2.86911	0.21698	0.24071
H	-2.32026	0.55103	2.87671
N	0.19394	0.3286	2.49836
C	1.58544	0.56435	2.10184
C	2.50353	-0.64417	2.41369
O	3.46381	-0.58002	3.17817
C	2.12587	1.8608	2.71607
C	1.42792	3.15021	2.24966
C	1.74542	3.61628	0.81251
N	1.12319	4.86815	0.3849
C	1.54636	6.09288	0.73929
N	2.61043	6.26656	1.54328
N	0.94196	7.17803	0.23631
H	-0.11231	0.68763	3.39437
H	1.57234	0.64943	1.0123
H	2.05902	1.78934	3.80925
H	3.19545	1.91624	2.48709
H	0.34318	3.05587	2.38712
H	1.74541	3.96287	2.91467
H	2.83245	3.70431	0.69549
H	1.41797	2.85045	0.10249
H	0.23765	4.82027	-0.15031
H	2.95792	5.49521	2.09187
H	2.86138	7.2285	1.84924
H	0.13009	7.04895	-0.3902
H	0.98959	8.02829	0.82034
N	2.18034	-1.75708	1.69469
C	3.06033	-2.90222	1.56955
C	3.87443	-2.93874	0.25987
O	4.47005	-3.9654	-0.07565
H	1.3697	-1.68922	1.08023
H	3.76011	-2.88787	2.40935
N	3.86844	-1.78599	-0.43853
C	4.46103	-1.58019	-1.7423
C	5.63168	-0.58967	-1.71427
O	6.0422	-0.01297	-2.7034
C	3.39224	-1.12824	-2.75191
C	2.23052	-2.13052	-2.94604
O	2.1741	-3.12887	-2.1543
O	1.43208	-1.87847	-3.88032
H	3.34788	-0.98419	-0.10181
H	4.88349	-2.5414	-2.05852
H	2.97236	-0.16866	-2.43374
H	3.86343	-0.96343	-3.72652

C	7.33497	0.39783	-0.41878
H	8.1349	0.04159	-1.07234
H	7.05789	1.41518	-0.70503
H	7.65034	0.36657	0.62365
O	6.19128	-0.47607	-0.50119
C	-7.84669	-3.3321	-0.84878
H	-8.86256	-3.02028	-1.11171
H	-7.56844	-4.14638	-1.52572
H	-7.84535	-3.7084	0.17771
H	2.49105	-3.83464	1.62288
H	-2.2962	-1.21039	2.787

Supplementary Table S1. NMR chemical shifts of the Hε, Cζ and Nε nuclei of arginine side chain in a model peptide with or without salt bridge, predicted through Density Functional Theory (DFT) calculations.

Arginine side-chain	with salt bridge	without salt bridge	Chemical shift change*
**Hε (ppm)**	9.847	5.632	-4.215
**Cζ (ppm)**	158.896	158.149	-0.747
**Nε (ppm)**	111.228	113.147	1.919
**Nη1 (ppm)**	92.864	93.089	0.224
**Nη2 (ppm)**	100.390	91.807	-8.583

*. Chemical shift change upon disruption of the salt bridge.

Supplementary Table S2. Frequency of Aβ40 conformers containing Arg5-based salt bridges, as obtained from analysis of a 30-μs long MD trajectory of Aβ40.[12] The frequencies are reported separately for various modes of interaction between the guanidinium and carboxylate groups.

	salt-bridge mode			
	**side-on**	**end-on**	**backside**	**total**
**R5-D7**	12.9%	0.3%	1.1%	14.4%
**R5-E3**	6.7%	1.6%	2.0%	10.3%
**R5-E11**	2.0%	1.4%	3.0%	6.4%
**R5-D1**	0.8%	0.6%	0.9%	2.3%

Supplementary References

1. Rezaei-Ghaleh, N.; Amininasab, M.; Giller, K.; Kumar, S.; Stundl, A.; Schneider, A.; Becker, S.; Walter, J.; Zweckstetter, M. Turn plasticity distinguishes different modes of amyloid-beta aggregation. *J. Am. Chem. Soc.* **2014,** *136*, 4913-4919.
2. Hou, L.; Shao, H.; Zhang, Y.; Li, H.; Menon, N. K.; Neuhaus, E. B.; Brewer, J. M.; Byeon, I. J.; Ray, D. G.; Vitek, M. P.; Iwashita, T.; Makula, R. A.; Przybyla, A. B.; Zagorski, M. G. Solution NMR studies of the A beta(1-40) and A beta(1-42) peptides establish that the Met35 oxidation state affects the mechanism of amyloid formation. *J. Am. Chem. Soc.* **2004,** *126*, 1992-2005.
3. Roche, J.; Shen, Y.; Lee, J. H.; Ying, J.; Bax, A. Monomeric Abeta(1-40) and Abeta(1-42) Peptides in Solution Adopt Very Similar Ramachandran Map Distributions That Closely Resemble Random Coil. *Biochemistry* **2016,** *55*, 762-775.
4. Delaglio, F.; Grzesiek, S.; Vuister, G. W.; Zhu, G.; Pfeifer, J.; Bax, A. NMRPipe: a multidimensional spectral processing system based on UNIX pipes. *J. Biomol. NMR* **1995,** *6*, 277-293.
5. Shen, Y.; Bax, A. Identification of helix capping and b-turn motifs from NMR chemical shifts. *J. Biomol. NMR* **2012,** *52*, 211-232.
6. Berjanskii, M. V.; Wishart, D. S. A simple method to predict protein flexibility using secondary chemical shifts. *J. Am. Chem. Soc.* **2005,** *127*, 14970-14971.
7. Munte, C. E.; Beck Erlach, M.; Kremer, W.; Koehler, J.; Kalbitzer, H. R. Distinct conformational states of the Alzheimer beta-amyloid peptide can be detected by high-pressure NMR spectroscopy. *Angew. Chem. Int. Ed. Engl.* **2013,** *52*, 8943-8947.
8. Koehler, J.; Erlach, M. B.; Crusca, E.; Kremer, W.; Munte, C. E.; Kalbitzer, H. R. Pressure Dependence of N-15 Chemical Shifts in Model Peptides Ac-Gly-Gly-X-Ala-NH2. *Materials* **2012,** *5*, 1774-1786.
9. Karunanithy, G.; Reinstein, J.; Hansen, D. F. Multiquantum Chemical Exchange Saturation Transfer NMR to Quantify Symmetrical Exchange: Application to Rotational Dynamics of the Guanidinium Group in Arginine Side Chains. *J. Phys. Chem. Lett.* **2020,** *11*, 5649-5654.
10. Guenneugues, M.; Berthault, P.; Desvaux, H. A method for determining B1 field inhomogeneity. Are the biases assumed in heteronuclear relaxation experiments usually underestimated? *J. Magn. Reson.* **1999,** *136*, 118-126.
11. Rezaei-Ghaleh, N.; Kumar, S.; Walter, J.; Zweckstetter, M. Phosphorylation Interferes with Maturation of Amyloid-beta Fibrillar Structure in the N Terminus. *J. Biol. Chem.* **2016,** *291*, 16059-16067.
12. Robustelli, P.; Piana, S.; Shaw, D. E. Developing a molecular dynamics force field for both folded and disordered protein states. *Proc. Natl. Acad. Sci. U. S. A.* **2018,** *115*, E4758-E4766.
13. Donald, J. E.; Kulp, D. W.; DeGrado, W. F. Salt bridges: geometrically specific, designable interactions. *Proteins* **2011,** *79*, 898-915.
14. M. J. Frisch, G. W. T., H. B. Schlegel, G. E. Scuseria, M. A. Robb, J. R. Cheeseman, G. Scalmani, V. Barone, B. Mennucci, G. A. Petersson, H. Nakatsuji, M. Caricato, X. Li, H. P. Hratchian, A. F. Izmaylov, J. Bloino, G. Zheng, J. L. Sonnenberg, M. Hada, M. Ehara, K. Toyota, R. Fukuda, J. Hasegawa, M. Ishida, T. Nakajima, Y. Honda, O. Kitao, H. Nakai, T. Vreven, J. A. Montgomery, Jr., J. E. Peralta, F. Ogliaro, M. Bearpark, J. J. Heyd, E. Brothers, K. N. Kudin, V. N. Staroverov, T. Keith, R. Kobayashi, J. Normand, K. Raghavachari, A. Rendell, J. C. Burant, S. S. Iyengar, J. Tomasi, M. Cossi, N. Rega, J. M. Millam, M. Klene, J. E. Knox, J. B. Cross, V. Bakken, C. Adamo, J. Jaramillo, R. Gomperts, R. E. Stratmann, O. Yazyev, A. J. Austin, R. Cammi, C. Pomelli, J. W. Ochterski, R. L. Martin, K. Morokuma, V. G. Zakrzewski, G. A. Voth, P. Salvador, J. J. Dannenberg, S. Dapprich, A. D. Daniels, O. Farkas, J. B. Foresman, J. V. Ortiz, J. Cioslowski, and D. J. Fox, Gaussian 09, Revision C.01. Gaussian 09, Revision C.01, M. J. Frisch, G. W. Trucks, H. B. Schlegel, G. E. Scuseria, M. A. Robb, J. R. Cheeseman, G. Scalmani, V. Barone, B. Mennucci, G. A. Petersson, H. Nakatsuji, M. Caricato, X. Li, H. P. Hratchian, A. F. Izmaylov, J. Bloino, G. Zheng, J. L.

Sonnenberg, M. Hada, M. Ehara, K. Toyota, R. Fukuda, J. Hasegawa, M. Ishida, T. Nakajima, Y. Honda, O. Kitao, H. Nakai, T. Vreven, J. A. Montgomery, Jr., J. E. Peralta, F. Ogliaro, M. Bearpark, J. J. Heyd, E. Brothers, K. N. Kudin, V. N. Staroverov, T. Keith, R. Kobayashi, J. Normand, K. Raghavachari, A. Rendell, J. C. Burant, S. S. Iyengar, J. Tomasi, M. Cossi, N. Rega, J. M. Millam, M. Klene, J. E. Knox, J. B. Cross, V. Bakken, C. Adamo, J. Jaramillo, R. Gomperts, R. E. Stratmann, O. Yazyev, A. J. Austin, R. Cammi, C. Pomelli, J. W. Ochterski, R. L. Martin, K. Morokuma, V. G. Zakrzewski, G. A. Voth, P. Salvador, J. J. Dannenberg, S. Dapprich, A. D. Daniels, O. Farkas, J. B. Foresman, J. V. Ortiz, J. Cioslowski, and D. J. Fox, Gaussian Inc., Wallingford CT, 2010.

15. Walker, M.; Harvey, A. J. A.; Sen, A.; Dessent, C. E. H. Performance of M06, M06-2X, and M06-HF Density Functionals for Conformationally Flexible Anionic Clusters: M06 Functionals Perform Better than B3LYP for a Model System with Dispersion and Ionic Hydrogen-Bonding Interactions. *J. Phys. Chem. A* **2013,** *117*, 12590-12600.

16. Zhao, Y.; Truhlar, D. G. The M06 suite of density functionals for main group thermochemistry, thermochemical kinetics, noncovalent interactions, excited states, and transition elements: two new functionals and systematic testing of four M06-class functionals and 12 other functionals. *Theor. Chem. Acc.* **2008,** *120*, 215-241.

17. Mardirossian, N.; Head-Gordon, M. Thirty years of density functional theory in computational chemistry: an overview and extensive assessment of 200 density functionals. *Mol. Phys.* **2017,** *115*, 2315-2372.

18. Weigend, F. Accurate Coulomb-fitting basis sets for H to Rn. *Phys. Chem. Chem. Phys.* **2006,** *8*, 1057-1065.

19. Weigend, F.; Ahlrichs, R. Balanced basis sets of split valence, triple zeta valence and quadruple zeta valence quality for H to Rn: Design and assessment of accuracy. *Phys. Chem. Chem. Phys.* **2005,** *7*, 3297-3305.

20. Tomasi, J.; Mennucci, B.; Cammi, R. Quantum Mechanical Continuum Solvation Models. *Chem. Rev.* **2005,** *105*, 2999-3094.

21. Stephens, P. J.; Devlin, F. J.; Chabalowski, C. F.; Frisch, M. J. Ab Initio Calculation of Vibrational Absorption and Circular Dichroism Spectra Using Density Functional Force Fields. *J. Phys. Chem.* **1994,** *98*, 11623-11627.

22. Vosko, S. H.; Wilk, L.; Nusair, M. Accurate spin-dependent electron liquid correlation energies for local spin density calculations: a critical analysis. *Can. J. Phys.* **1980,** *58*, 1200-1211.

23. Becke, A. D. Density-functional thermochemistry. III. The role of exact exchange. *J. Chem. Phys.* **1993,** *98*, 5648-5652.

24. Lee, C.; Yang, W.; Parr, R. G. Development of the Colle-Salvetti correlation-energy formula into a functional of the electron density. *Phys. Rev. B* **1988,** *37*, 785-789.

25. Hehre, W. J.; Ditchfield, R.; Pople, J. A. Self—Consistent Molecular Orbital Methods. XII. Further Extensions of Gaussian—Type Basis Sets for Use in Molecular Orbital Studies of Organic Molecules. *J. Chem. Phys.* **1972,** *56*, 2257-2261.

26. Ditchfield, R. Self-consistent perturbation theory of diamagnetism. *Mol. Phys.* **1974,** *27*, 789-807.

27. Adamo, C.; Barone, V. Exchange functionals with improved long-range behavior and adiabatic connection methods without adjustable parameters: The mPW and mPW1PW models. *J. Chem. Phys.* **1998,** *108*, 664-675.

28. Perdew, J. P.; Chevary, J. A.; Vosko, S. H.; Jackson, K. A.; Pederson, M. R.; Singh, D. J.; Fiolhais, C. Atoms, molecules, solids, and surfaces: Applications of the generalized gradient approximation for exchange and correlation. *Phys. Rev. B* **1992,** *46*, 6671-6687.

29. CHESHIRE (chemical shift repository with coupling constants added too, http://cheshirenmr.info/)
